# On sample preparation methods for fermented beverage VOCs profiling by GCxGC-TOFMS

**DOI:** 10.1007/s11306-020-01718-7

**Published:** 2020-09-19

**Authors:** Penghan Zhang, Silvia Carlin, Cesare Lotti, Fulvio Mattivi, Urska Vrhovsek

**Affiliations:** 1 Metabolomic Unit, Food Quality and Nutrition Department, Research and Innovation Center, Edmund Mach Foundation, Via E. Mach 1, 38010 San Michele all’Adige, Italy; 2 Department of Cellular Computational and Integrative Biology (CIBIO), University of Trento, Via Sommarive 9, 38123 Povo, Trento, Italy

**Keywords:** Fermented beverages, VOC profiling, Sample preparation methods, Two dimensional gas chromatography–mass spectrometry

## Abstract

**Introduction:**

Aromas and tastes have crucial influences on the quality of fermented beverages. The determination of aromatic compounds requires global non-targeted profiling of the volatile organic compounds (VOCs) in the beverages. However, experimental VOC profiling result depends on the chosen VOC collection method.

**Objectives:**

This study aims to observe the impact of using different sample preparation techniques [dynamic headspace (DHS), vortex-assisted liquid–liquid microextraction (VALLME), multiple stir bar sorptive extraction (mSBSE), solid phase extraction (SPE), and solid phase micro-extraction (SPME)] to figure out the most suitable sample preparation protocol for profiling the VOCs from fermented beverages.

**Methods:**

Five common sample preparation methods were studied with beer, cider, red wine, and white wine samples. After the sample preparation, collected VOCs were analyzed by two-dimensional gas chromatography coupled with time of flight mass spectrometry (GCxGC-TOFMS).

**Results:**

GCxGC oven parameters can be optimized with the Box–Behnken surface response model and response measure on peak dispersion. Due to the unavoidable column and detector saturation during metabolomic analysis, errors may happen during mass spectrum construction. Profiling results obtained with different sample preparation methods show considerable variance. Common findings occupy a small fraction of total annotated VOCs. For known fermentative aromas, best coverage can be reached by using SPME together with SPE for beer, and VALLME for wine and cider.

**Conclusions:**

GCxGC-TOFMS is a promising tool for non-targeted profiling on VOCs from fermented beverages. However, a proper data processing protocol is lacking for metabolomic analysis. Each sample preparation method has a specific profiling spectrum on VOC profiling. The coverage of the VOC metabolome can be improved by combining complementary methods.

**Electronic supplementary material:**

The online version of this article (10.1007/s11306-020-01718-7) contains supplementary material, which is available to authorized users.

## Introduction

Aromas and tastes are the major factors influencing the consumer’s perception of the quality of the wine, beer, and other fermented beverages (García-Muñoz et al. 2014). Determining the aromatic compounds in those beverages and understanding their effects on the human organoleptic effect is important (Humia et al. 2019). The determination of aromatic compounds usually starts with the characterization and quantification of the volatiles (Pinu 2018). In the past, due to the insufficient analytical capability, understanding the flavor perception was supported by models with simplified chemical inputs. Important volatile compounds were preselected based on their odor activity values (OAVs) (Patton and Josephson 1957). Widely used approaches are aroma extract dilution analysis (AEDA) and odor-specific magnitude estimation (OSME) (Ullrich and Grosch 1987; McDaniel et al. 1990). According to the OAVs, about 75 aromatic compounds were found and listed in a review study (Cullere et al. 2019).

With the development of modern chromatography, potential aromatic compounds can be selected based on the metabolomic scenario (Flavoromics – the next frontier? 2008). During this approach, a wider spectrum of chemical compounds are studied by chemometrics tools, aimed to explore the chemical composition of food to its sensory property. With the potential to reveal previously ignored aromatic compounds and relationships. Eventually, a better perception model can be constructed. To apply a chemometric approach, complete profiling of volatile organic compounds (VOCs) is desirable. One of the challenges on the mapping work is that the number of volatile compounds in a fermented beverage is over 1300 (Nykänen and Suomalainen 1983). An advanced analytical platform is demanded to detect and ideally identify each of them. Two-dimensional gas chromatography coupled with a mass spectrometer (GCxGC-MS) might be a solution (Cordero et al. 2015). However, the results of VOC profiling are not only separation and detection dependent, but also sample preparation dependent (Stefanuto et al. 2017). Unfortunately, because of the diversity of VOCs, an ideal method that can concentrate and recover all VOC compounds does not exist. Commonly used sample preparation techniques for VOC analysis are dynamic headspace (DHS), Vortex-assisted liquid–liquid microextraction (VALLME), multiple stir bar sorptive extraction (mSBSE), solid phase extraction (SPE), and solid phase microextraction (SPME).

DHS is widely applied for the advantages as environment-friendly, easy to implement and versatile procedure. It uses a continuous flow of inert gas to extract the volatile organic compounds from a liquid matrix. Extracted VOCs are further concentrated into an adsorbent or cryogenic trap. In the ideal case, the signal intensity of an analyte is negatively linearly correlated to its remaining concentration in the sample, which depends on its original concentration and decreases exponentially with sampling time and evaporative constant (Kolb and Ettre 1991). Traps made of Tenax TA sorbent are the most common, owing to their wide absorption range, high-temperature stability, low water affinity, and long shelf life (Soria et al. 2015).

VALLME draws attention by its simplicity, speed, miniaturization and low-cost (Psillakis and Kalogerakis 2002). During the sample preparation, the sample and the organic phase are mixed together. Mass transfer of an analyte from sample to organic solvent phase depends on the product of the overall mass transfer coefficient on the interface and the interfacial area. By vortexing, a turbulence flow is generated to breakdown the solvent phase into small droplets, forming an emulsion. Consequently, the interfacial area is enlarged (Davies 2012). The emulsions formed during VALLME are thermodynamically unstable. At the final stage of sample preparation, according to Stoke's Law, a higher gravitational acceleration is introduced to speed up the decomposition of emulsion to form separate phases (Gupta et al. 2016).

The mSBSE and SPME are appreciated for their features of solvent-less extraction, predictable extraction efficiencies, multiple uses for determinations in gaseous and aqueous matrices, and excellent repeatability and reproducibility. They also share the same basic principle. VOCs are extracted from a sample based on their affinity for and solubility in a viscous liquid phase. The extraction is a kinetic process with theoretical recovery depending on the phase ratio and distribution coefficient. Certain sampling time is required to reach the final equilibrium state. One of the most important factors for mSBSE and SPME analysis is sorbent. Thanks to the low glass transition temperature (< 40 °C), very low bleeding profile at high temperatures (> 350 °C) after appropriate conditioning, and excellent diffusivity and permeability, PDMS is by far the best material for non-polar analytes (Baltussen et al. 2002). To cover more polar analytes in one analysis, supplementary sorbents are used in combination with PDMS. For SBSE, the only commercially available product is an ethylene glycol modified silicone material (EG silicon) (David et al. 2019). For SPME, the most common three-phases sorbent is DVB/Car/PDMS (Bojko et al. 2014).

Solid-phase extraction (SPE) is the most widely used and flexible method for the extraction, changing of solvents, clean-up, concentration and fractionation of organic compounds from several samples. SPE can absorb a wide range of organic analytes (from non-polar to very polar analytes) and has high extraction efficiency. Besides, analytes absorbed onto the SPE cartridge/column/disc are stable, can be stored for a long time (Andrade-Eiroa et al. 2016). The retention and elution mechanism of SPE is similar to the reverse-phase high-performance liquid chromatography. Solid-phase retains the molecule in three measures: partition for small nonpolar analytes (Martire and Boehm 1983), adsorption for larger nonpolar molecules (Gritti and Guiochonab 2006), and electronic interactions, such as hydrogen bonding, ionic interactions, or π–π bonds for polar and polarizable analytes (Pap et al. 2002). Many sorbents are available for SPE analysis. Among all, ENV+, hyper-crosslinked hydroxylated polystyrene divinylbenzene copolymer, shows high specific surface area and an extraordinary adsorption capability (Castaldo et al. 2017).

Different sample preparation techniques have been studied based on the analytical results obtained from mono-dimensional GC–MS (Liu et al. 2018; Picard et al. 2018). In some studies, GCxGC-MS have been applied to fermented alcoholic samples analysis with one or more sample preparation techniques (Carlin et al. 2016; Stefanuto et al. 2017). However, so far, to our knowledge, there has been no study on selecting the most suitable sample preparation techniques, coupled with untargeted GCxGC-MS, for the measurement of VOCs from fermented beverages. We studied the above mentioned sample preparation techniques followed by GCxGC-MS analysis to compare their capability of recovering VOCs from different fermented beverages (beer, white and red wine and cider).

## Material and methods

### Samples, reagents and supplies

Four types of fermented beverages, white wine, red wine, cider, and beer were studied. For each type of beverage, a pooled sample was prepared by mixing commercial products from local markets. Pooled white wine was obtained by mixing Riesling, Müller, Manzoni Bianco, Sauvignon blanc, Veltliner, and Gewürztraminer to cover as many volatile compounds as possible. Pooled red wine was made from single variety Blaufrankisch of different quality levels. Pooled cider sample covers various varieties from Trentino Alto Adige (Golden Delicious, Stark Delicious, Granny Smith, Royal Gala, Winesap, Morgenduft, Fuji, and Braeburn). Pooled beer was made of pale lager beers produced by Heineken. Beer samples were degassed by sonication for 10 s before the mixing.

To optimize the GCxGC separation, a stock solution of 10 mg/L in ethanol was prepared. It consists of 131 chemical compounds which are commonly present in fermented beverages (Table S1). Alkane standards (C9 to C30), internal standards (2-octanol and 1-heptanol), sodium chloride, ammonium sulfate, and dichloromethane were purchased from Sigma Aldrich.

### Sample preparation

#### Dynamic headspace

Tenax TA sorbent tube was conditioned at 315 °C for 1 h in TDS Tube Conditioner (Gerstel). Two milliliters of the sample were dosed into a 20 mL headspace vial which contains 2 g of NaCl. Before the sampling, 25 μl of 2-octanol solution (2 mg/L in ethanol) was added as internal standard. DHS sampling was carried out with Gerstel MPS2 auto-sampler (Mülheimander Ruhr, Germany), including the sampling, thermal desorption (ThermoDesorption Unit, Gerstel) and cryo focusing (Cooling Injection System, Gerstel). Detailed experimental parameters are listed in Table S 2.

#### Vortex-assisted liquid–liquid microextraction

The method was in accordance with a previous study (Ortega et al. 2001) with minor modification. 2.5 mL of sample was placed together with 3.5 mL deionized water and 2.5 g (NH_4_)_2_SO_4_ in a 15 mL glass vortex tube. After completely dissolve, 20 μL of internal standard solution (100 mg/L 1-heptanol in EtOH) and 0.2 mL of dichloromethane were added. The mixture was vortexed at 3200 rpm for 1 min and then centrifuged at 5 °C, 1174 RCF for 10 min. Once the phases were separated, the organic phase was transferred into a 0.3 mL vial by a 250 μL syringe (Hamilton). Collected volatile extracts were stored at − 20 °C until the analysis. From each extract, 1 μL was taken for the GCxGC analysis. Liquid injections were performed with Gerstel MPS2 auto-sampler monitored by ChromaToF (Leco, St Joseph, MI, USA).

#### Multiple stir bar sorptive extraction

Stir bars (Twister) coated with 24 μL PDMS and 32μL EG Silicone were soaked in acetonitrile for 12 h and then thermal conditioned by Gerstel TDS Tube Conditioner. PDMS stir was conditioned at 300 °C in a nitrogen flow for 60 min. For EG Silicone stir bar, the conditioning temperature and time were 220 °C and 30 min. After conditioning, they were placed in a 10 mL headspace vial (Ochiai et al. 2013). 10 mL of sample was dosed and sampled at room temperature for 1 h. The stirring speed was 800 rpm. After the sampling, the stir bar was washed by deionized water, dried with lint-free tissue and place in thermal desorption liner. Thermal desorption and cryo-focusing parameters are shown in Table S 3.

#### Solid phase extraction

Sample preparation and extraction protocols were modified from a previous study (Vrhovsek et al. 2014). ENV + cartridges (1 g Biotage, Sweden), used during solid-phase extraction, were pre-conditioned with 15 mL methanol followed by 20 mL of deionized water. For each sample, 50 mL of the sample (25 mL for degassed beer sample) was mixed with 100 μL n-heptanol solution (250 mg/L in ethanol) and loaded in the SPE cartridge. After the extraction, the solid phase was washed with 15 mL of deionized water. Analytes were recovered by 30 mL of dichloromethane and then concentrated to 250 μL.

#### Solid-phase microextraction

SPME fiber of 2 cm (50/30 DVB/CAR/PDMS), from Supelco, was conditioned according to the manual before use. For each pooled sample, 1 mL of the matrix was mixed with 1 g NaCl in a 20 mL headspace vial. Before the analysis, 25 μL of internal standard solution (2-octanol in ethanol at concentration of 2 mg/L) was added. Instrumental setup can be found in Table S 4. Other details can be found in the literature (Carlin et al. 2016).

### GCxGC separation and detection

GCxGC system was the Agilent 7890 A (Agilent Technologies, Santa Clara, CA). Injections were performed with splitless mode. Equipped columns were VF-Wax column (100% polyethylene glycol; 30 m × 0.25 mm × 0.25 μm, Agilent J&WScientific Inc., Folsom, CA) as the 1st dimension and Rxi-17Sil MS 1.50 m × 0.15 mm × 0.15 μm, Restek Bellefonte, USA) as the 2nd dimension. A non-moving quad jet dual-stage thermal modulator was used to coupling the two columns. MS signal was obtained with Pegasus IV time-of-flight mass spectrometer (Leco Corporation, St. Joseph, MI).

GCxGC separation parameters were optimized according to the Box–Behnken experiment design (JMP, SAS Institute, Cary, NC, USA). Used column setup was a polar column followed by a medium polar column, recommended by a previous study (Welke et al. 2012).Considered independent variables were column flow, temperature program, 2nd oven temperature offset, modulation temperature offset, modulation time, hot pulse time (% of the entire modulation time). The dependent variable was the median value of Nearest Neighbor Distance (NND) (Nowik et al. 2013), which is calculated only based on the annotated peaks. Modulation time was adjusted to avoid the wrap around phenomena. After the optimization, same standard solution was injected and separated under the model suggested condition to confirm the real separation measure.

The optimized column flow was 0.8 mL/min. The oven temperature was a program from 40 °C (2 min holding time) to 250 °C at a rate of 3 °C/min. After reaching the final temperature of 250 °C, 15 min holding time was applied. The temperature offsets of second oven and modulator were fixed at + 5 °C and + 8 °C respectively. Within the 6-s-modulation time, 2.1 s are used for hot pulse. The transfer line was kept at 250 °C. The TOFMS was operated in electron ionization mode at 70 eV. The ion source temperature was 230 °C. The acquisition frequency was 200 Hz within a mass scan range from 35 to 450 m/z. The detector voltage was − 1341 V. In the case of the liquid injection, an acquisition delay of 7 min was applied. This setup was applied to the measurements of all types of beverages.

### Data processing and peak annotation

GCxGC-MS data acquisition and processing were achieved with LECO ChromaTOF (Version 4.22). The processing consists of peak picking, peak annotation, and statistic confirmation. During the peak picking, signals which were just above the noise were taken into account (baseline offset = 1). Minimal expected peak width (on 2nd dimension) for deconvolution was 0.8 s. A peak was defined when at least five ions whose signal to noise ratio is above 100 (Stefanuto et al. 2017), can be grouped. A picked peak was annotated by matching its mass spectrum (MS) to the reference spectrum in the database. In this study, used MS databases were NIST/EPA/NIH 11, Wiley 8 and the FFNSC 2. The MS similarity threshold for the peak annotation was 700. To determine this value, a standard MS library was created by recording the real measured mass spectrums of chemical standards (Table S 1) under a well-separated condition. The same chemicals were mixed and analyzed under different GCxGC separation conditions (Table S 5). A reversed match mode (match the library mass spectrums to the measured mass spectrum) was used to identify these chemical peaks. The similarity of the mass spectrum of each peak under each separation condition was collected and to be used to evaluate the similarity threshold value. For pooled samples, to minimize the peak detection and annotation error, each sample was analyzed with three technical replications. Inter-measurements peak alignment was performed based on the retention times (both 1st and 2nd dimensions) and mass spectrum. A minimal MS similarity of 600 was required. An analyte was further examined, only if it can be detected in all the technical replications. An inter-class comparison was performed between sample class and blank class. Fisher ratio thresholding was used to eliminate artifact compounds (sorbent bleeding, column bleeding and other possible interferences). The applied significance level was 0.05. Peak identification was then completed by checking the linear temperature programmed retention index (LTPRI), which is available in the NIST RI database.

## Result and discussion

### Separation optimization

Many methods have been published to measure the separation degree for two-dimensional chromatography. A brief review of each type of method was published in a previous study (Mommers and Wal 2019). Among all the methods, nearest neighbor distance was chosen because it measures the absolute dispersion among all the peaks. For non-targeted profiling, sample composition is usually complex and often requires an extended analytical time. If the separation space is limited (in the time domain), approaches based on the orthogonality measure are more suitable. All the major oven parameters for GCxGC separation were taken into account during the optimization (Table S 5).

The experimental data were analysis by a Box–Behnken surface response model, which allows efficient estimation of the first- and second-order coefficients for input variables (Bezerra et al. 2008). The model is perfectly fitted with adjust R^2^ is nearly 1 (Table S 6). Apart from the quadratic term made of column flow and temperature program rate, all the model components have significant influences on the separation result. The model supposes that the oven temperature program rate and modulation time are the most important parameters (Tables S 7 and S 8). This result is expected. Oven temperature directly links to the retention time, which linearly impacts the separation resolution. Modulator act as a collector for elute compounds. Elongate the modulation time reduces the separation resolution of the 1^st^ dimension. A lower value for oven temperature and modulation time is always desired unless the wrap-around effect happens. When a compromise has to be made, according to the absolute value of estimates of each parameter, higher priority has to be given to the temperature rate. Other parameters were optimized within the range in which a valid chromatogram can be obtained (Table S 5). Chosen values are not always at apices. Optimized separation parameters were: Column flow, 0.8 mL/min; Modulation time, 6 s; 2nd oven temperature offset, 5 °C; Temperature program, 3 °C; Modulation temperature offset, 8 °C; Hot pulse time, 35% of the entire modulation time. Model predicted median NND under the optimized oven condition was 14.6 s (Fig. S 1). The experimental confirmation shows the true median NND was 12 s, which is 20% lower than the model predicted value.

### MS similarity

MS matching is the most important step for peak annotation. A suitable similarity threshold value should concern both the annotation accuracy and recovery of the not-well-resolved peaks. A previous study (Bean et al. 2015) recommended using a moderate MS matching score, between 500 and 700, to obtain the highest proportion of reproducible peak. In our study, a standard solution, which contains 131 common volatile compounds from wine, was analyzed under the diverse separation conditions. Sum-up the analytical results under all the separation conditions, 2715 annotation were made by reserved matching. Matching scores of each annotation were collected and the distribution is shown in Fig. S 2. The majority of the peaks, 2149 out of 2715, matched to the library mass spectrum with a relatively high score (> 800). Below 800, peak identification was improved by 2‰ for every 20 unit reduction on matching score. Eventually, a sharp drop in the improvement was observed starting from 720. It is reasonable to set the similarity threshold value at 700.

The deviation of the MS is the result of peak picking error which is caused by over deconvolution. As demonstrated in Fig. S 3. The MS signals of 1-phenylethyl acetate were grouped into two peaks. The combination of the constructed two mass spectra is more similar to the reference mass spectrum from the NIST library. Over deconvolution can be partially eliminated—in theory—by preventing the peak from the non-Gaussian shape and increasing the expected peak width value during the data processing. However, in the case of the non-targeted metabolomics study, the content of the compounds can vary up to 10^6^ magnitudes. It’s not possible to find a sample preparation approach that allows the presence of trace components in the chromatogram, and at the meantime, keeps the peak shapes Gaussian-like for major compounds; or define an ideal peak width for the entire chromatogram. Tolerance from the MS matching score is necessary.

**Fig. 1:**
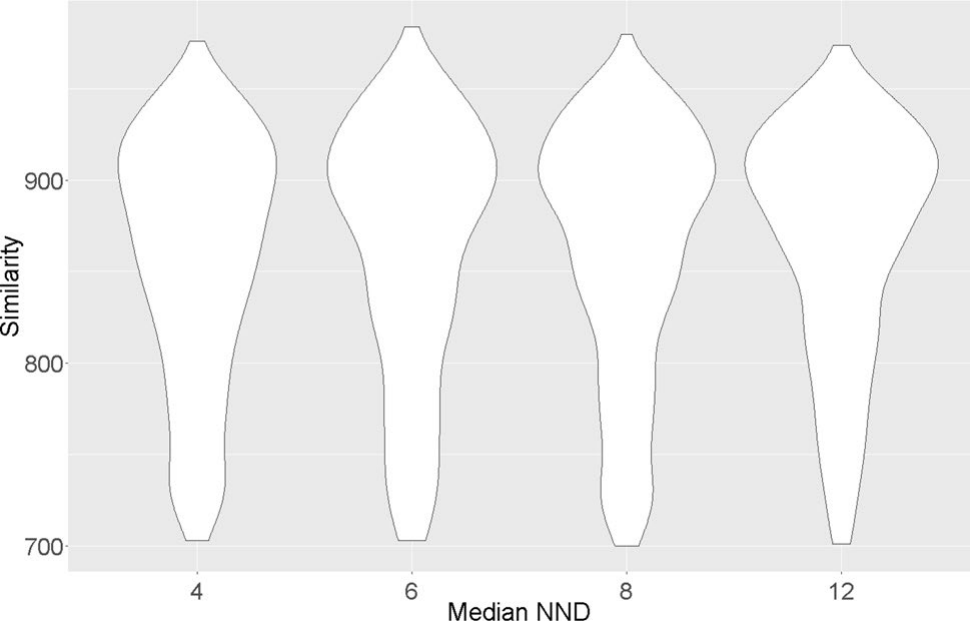
Violin plot of similarity distribution and Median NND

With a better separation (Sect. 3.1), peaks are better picked. Consequently, the constructed mass spectrum should be more similar to the reference spectrum in the library. A violin plot (Fig. 1) was created to visualize the relationship between the similarity distribution and the median of nearest neighbor distance, which is a global measure of peak separation (Nowik et al. 2013). From 4 to 12 NND units, peaks are more likely to have the MS matching score at 900 and less likely to be at 700. However, the improvement is not remarkable. A reason could be that the improvement of the global separation measure is not equivalent to the better separation at the non-well separated region. Compared to a real natural sample, the standard solution used for the test only contains a limited number of compounds. A threshold value would be lower than 700 if peaks are heavily overlapping with each other. There is still some room for the improvement on the peak picking. A better data processing protocol would be desired for the complex sample measurement in the metabolomics study.

### Comparing the analysis of five sampling techniques on four types fermented beverages

On pooled beer, red wine, cider, and white wine sample, DHS, VALLME, mSBSE, SPE and SPME sampling techniques were tested. VALLME does not apply to the beer sample, since after sample preparation, the organic phase is mixed with a gel phase. It’s impossible for the further GC injection.

Only considering the annotated peak number, the best technique for the volatile compounds profiling was: SPE for beer (166) and red wine (433), DHS for cider (330) and VALLME for white wine (256). Details are listed in Table 1. If the automation level is taken into account, SPME would be the best choice for the beer sample, because SPE can only be operated manually. The experimental properties of each sample preparation technique are presented in Table 2.

**Table 1:** Numbers of annotated peaks obtained from different sample preparation techniques

	DHS	VALLME	mSBSE	SPE	SPME
Pooled beer	53	N.A	130	166	142
Pooled red wine	257	303	239	433	150
Pooled cider	330	257	272	280	223
Pooled white wine	215	256	168	218	145

**Table 2:** Comparison of five sampling techniques

	DHS	VALLME	mSBSE	SPE	SPME
Sample quantity	2 ml	2.5 ml	10 ml or 1 ml (10x^a^)	50 ml (10x^a^)	1 ml
Preparation time	≈ 30 min	≈ 10 min	≈ 1 h	≈ 2 h	≈ 20 min
Labor cost	Auto	Manual	Manual	Manual	Auto
Drawbacks	Need opt. for different sample	Not applicable for beer sample	Incomplete thermal desorption	Solvent and time cost	

^a^10 times dilution (see Sects. 3.4 and 3.5)

**Fig. 2:**
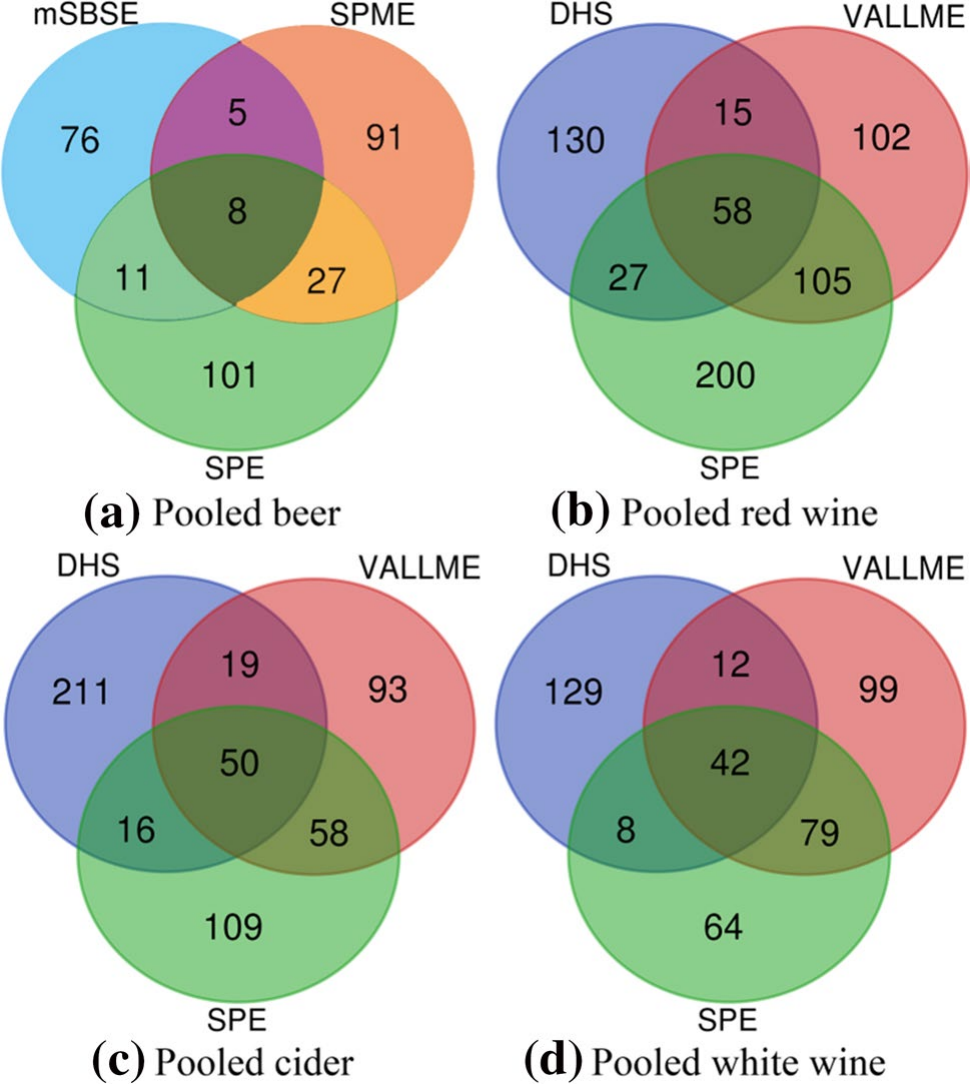
Venn plot for the selected best three sample preparation techniques on four types of fermented beverages

For each type of sample, the best three techniques are further compared by the Venn Plot (Fig. 2). Surprisingly, each technique has its own unique profiling spectrum. Only a small fraction of the annotated peaks can be found by all three techniques. On the other hand, this means that a combination of different sample preparation techniques has the potential to improve global coverage dramatically. To be noticed, the data presented here are not all the features contained in the chromatogram but only the annotated ones.

**Fig. 3:**
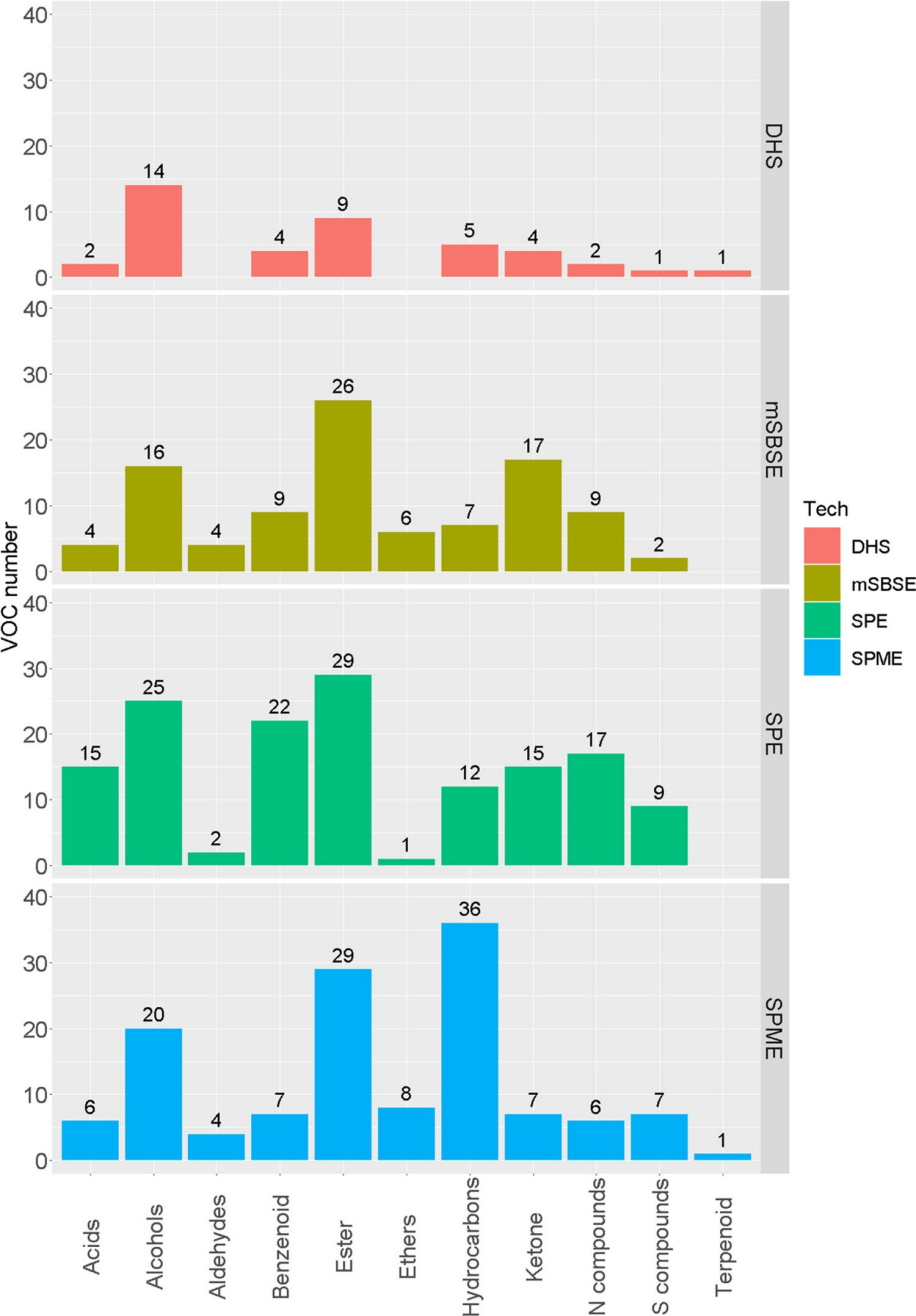
Comparison of the aligned peak number by chemical classes for applying different sample preparation techniques on pooled beer

To have a more detailed look, for each technique, identified compounds were classified according to their chemical categories. Bar plots were generated for beer (Fig. 3), red wine (Fig. S 4), cider (Fig. S 5), and white wine (Fig. S 6). Taking the measurements on beer as an example: SPE has better sampling performance for the volatile compounds in most chemical classes, except ethers and hydrocarbons (including terpenes). SPME is good at collecting and concentrating hydrocarbons (including terpenes), esters, and alcohols. DHS, probably, is not an ideal technique for VOCs profiling for beer. For the red wine sample, with SPE, fewer peaks were found in aldehyde class and in ether class compared to DHS and mSBSE respectively. It’s hard to reach an agreement on which technique or even the combination of the techniques is the most suitable for the VOCs profiling on red wine, cider, and white wine. The choice will be easier to make with a specific study focus.

### SPE sample dose optimization

For the non-target profiling analysis, analytical techniques and protocols which allow higher sample dose, are preferred to concentrate the trace compounds above the detection limit of the instrument. Taking into consideration that several key odorants usually present at trace levels (ng to µg/L) are recognized by the human olfactory system. However, in the case of the two dimensional GC separation followed by automatic peak picking and annotation, drawbacks of the higher sample dose must be noticed. The influence of peak intensity and resolution on alignment has been studied before (Bean et al. 2015). A negative result will be obtained when major chemicals saturate the column or the detector. Besides, overdose interferes with the measurement of trace compounds by decreasing the overall peak resolution. SPE was performed with 50 mL of pooled red wine sample. To study the influence of sample dose, a dilution series was made from 10 to 1000 times. With 100 times dilution, most inter-measurement aligned features (938) were detected. With 10 times dilution, only 30 peaks can be aligned. A possible strategy to improve sampling would be: use relatively less quantity of sample, make a dilution to reach the quantity demanded by the protocol and use excessive sorbent or a combination of different sorbents to increase the recovery.

### mSBSE limitations and potential

Compare to SPME, the performance of mSBSE is not very satisfying. For most chemical classes, the peak number found with mSBSE is only slightly higher than the SPME. If we look at hydrocarbons measurement on pooled beer (Fig. 3), more compounds were found by SPME (36) than mSBSE (7). However, in both techniques, PDMS was used to collect the non-polar compounds. The amount of sorbent coated on the stir bar is at least 50 times higher than the amount on SPME fiber. Besides, the amount of sample used during the SPE sampling was 2.5 times (adjusted by dilution before the GC injection) of SPME. A possible explanation for fewer peaks found with mSBSE sampling is there was incomplete thermal desorption for PDMS stir. According to the manual, the recommended thermal desorption (TD) temperatures for PDMS stir is 300 °C. However, during desorption, the temperature can rise until 220 °C, limited by the thermal tolerance of EG silicon stir. The retaining effect was confirmed by a second injection after the TD-GCxGC-MS analysis (Fig. S 7). To obtain better desorption, two types of stirs can be injected separately. This solution is not applicable to the analysis with two dimensional GC. Because of automatic peak annotation requires technical replications on one sample for applying fisher ratio thresholding. Even with three as minimum number of replication, a separate thermal desorption for two types of stir bars means six times TD-GCxGC-MS measurements for a single sample. The time cost is extremely expensive. However, the application potential of mSBSE cannot be denied. Even with the incomplete desorption, more compounds can be found with mSBSE sampling compare to SPME. The final state of the sampling is equilibrium. It means that the optimization for mSBSE is easier than DHS where breakthrough effect takes place. The protocol of mSBSE is less complex than VALLME and SPE.

### Determine the fermentative aromatic compounds

**Fig. 4:**
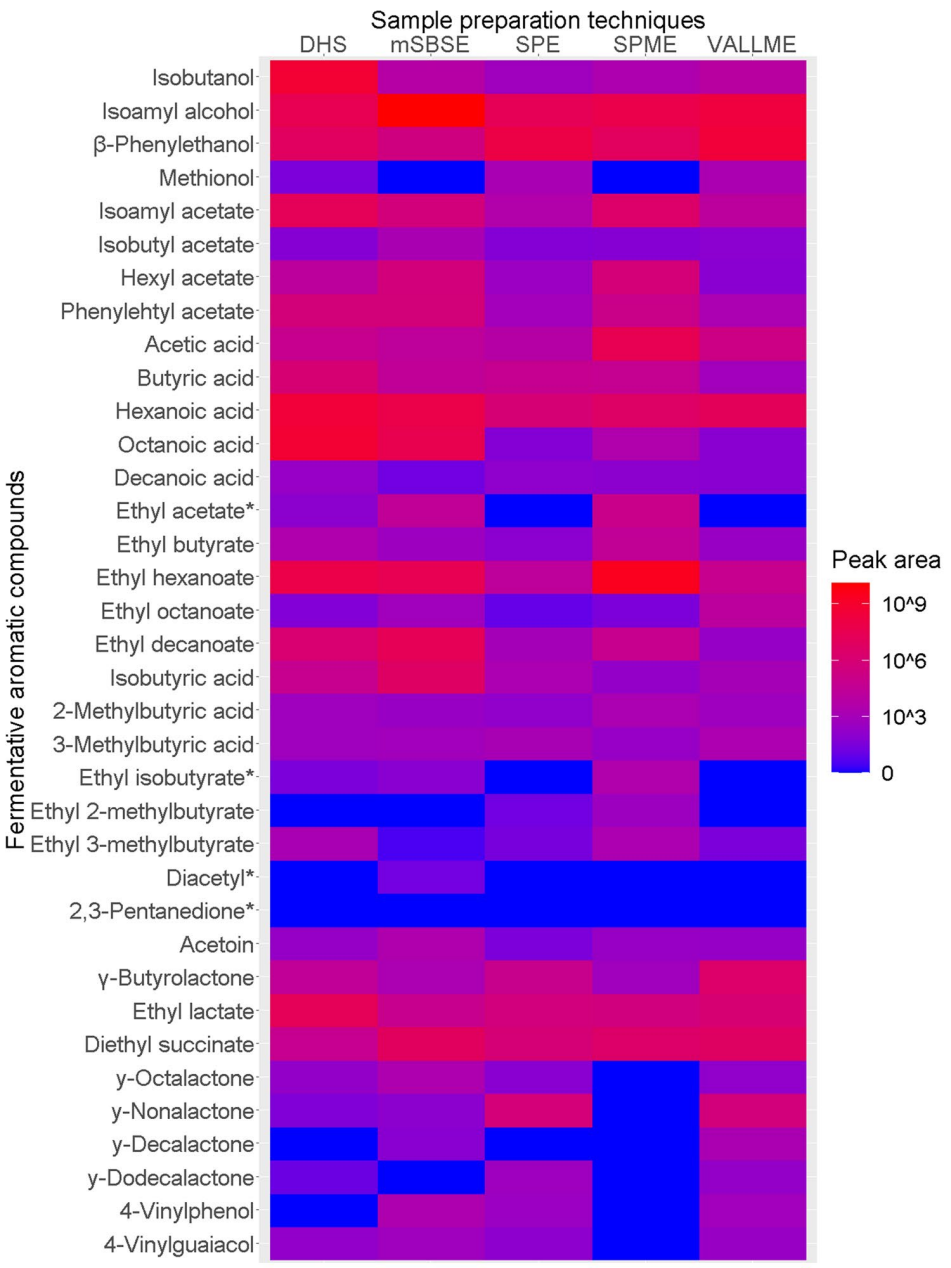
Heat plot for fermentative aromatic compounds determination with different sampling techniques on pooled white wine

Important fermentative aromatic compounds in beverages have been reviewed by Ferreira and his group (Cullere et al. 2019). According to this study, the analytical capability of each sample preparation technique followed by GCxGC-MS analysis has been evaluated. Each technique was applied to four types of beverages to check if targeted aromatic compounds can be found and how much signal intensity (peak area) can be obtained. Heat maps were used to visualize the result for white wine (Fig. 4), beer (Fig. S 8), red wine (Fig. S 9), and cider (Fig. S 10). Based on the result of white wine (Fig. 4), most aromatic compounds can be found with all the techniques. Comparing the most convenient technique SPME to other techniques, SPME is stronger on determining butyric esters and weaker on determining lactone and vinyl containing VOCs. If liquid–liquid extraction is applicable, extracting the sample with SPME and VALLME can reach the maximum analytical coverage on those fermentative aromatic compounds. In the beer case, when VALLME is not applicable, SPME together with SPE will provide the maximum analytical coverage. For earlier elutes, such as ethyl acetate, ethyl isobutyrate, diacetyl, 2,3-pentanedione, solvent delay time must be carefully set. The automatic annotation for lactone containing compounds was not successful. Manually annotation was performed. The difficulty is that many lactone-containing compounds have similar mass spectrums. Using a forward MS matching strategy will easily result in misidentification. Besides, one lactone compound has dozens of names. Even with a correct annotation, linking the result to what is looking for is difficult. A possible solution would be defining a library of interesting compounds, process the reverse matching according to the retention index of the selected compounds.

## Conclusion

GCxGC-TOFMS is an efficient tool for the analysis of volatile compound. GCxGC oven separation can be efficiently optimized by response surface model. In the case of non-target profiling for complex metabolic samples, the nearest neighbor distance was a suitable measurement for peak dispersion. When the column and detector were saturated by the major metabolites, which is unavoidable if the metabolic sample is measured at one dilution level, incorrect peak deconvolution and mass spectrum construction may happen. This limited the application of GCxGC-TOFMS to metabolites screening. Different sample preparation techniques were compared based on the results of VOC collection for fermented beverages. There wasn’t an ideal sample preparation technique to recover all the VOCs from the sample. Furthermore, the VOCs recovered by different techniques were very different. The discussion of the pros and cons of the different techniques in our study can serve as a guide for the further development and improvement of these techniques. Combining the results from different sample preparation techniques is necessary to achieve a higher coverage of global VOC profiling. For the known fermentative aromatic compounds, the best coverage can be reached by using SPME together with SPE for beer, and VALLME for wine and cider.

## Electronic supplementary material

Below is the link to the electronic supplementary material.

Supplementary file1 (DOCX 671 kb)

## Data Availability

The metabolomics and metadata reported in this paper are available via [MetaboLights (https://www.ebi.ac.uk/metabolights/)] study identifier [MTBLS1660].
