# Supplementary material for: On sample preparation methods for fermented beverage VOCs profiling by GCxGC-TOFMS
Source: Metabolomics. 2020 Sep 19;16(10):102. doi: 10.1007/s11306-020-01718-7 (PMC7502039; doi:10.1007/s11306-020-01718-7)

# Supplementary

Table S 1: Standard solution of 131 common aromatic compounds

| LTPRI | Supplier | Compound | CAS | Unique Mass | Classes |
| --- | --- | --- | --- | --- | --- |
| 1003 | Fluka | 2-butanol | 78-92-2 | 59 | alcohol |
| 1033 | Aldrich | butanoic acid, ethyl ester | 105-54-4 | 71 | ester |
| 1042 | Fluka | α-pinene | 127-91-3 | 93 | terpenes |
| 1084 | Fluka | 2-hexanone | 591-78-6 | 77 | ketone |
| 1092 | SAFC | *trans*-2-methyl-2-butenal | 497-03-0 | 84 | aldehyde |
| 1100 | Aldrich | 3-methyl-2-butanol | 26184-62-3 | 55 | alcohol |
| 1112 | Fluka | 3-pentanol | 584-02-1 | 59 | alcohol |
| 1115 | Supelco | n-butanol | 71-36-3 | 59 | alcohol |
| 1116 | Fluka | β-pinene | 127-91-3 | 93 | terpenes |
| 1119 | SAFC | 2-methylbutyl-acetate | 624-41-9 | 70 | ester |
| 1119 | Fluka | isopentyl acetate | 123-92-2 | 70 | ester |
| 1130 | SAFC | 2-methyl-2-pentenal | 623-36-9 | 98 | aldehyde |
| 1138 | Fluka | ethyl valerate | 539-82-2 | 85 | ester |
| 1139 | Aldrich | propanoic acid, butyl ester | 590-01-2 | 75 | ester |
| 1171 | SAFC | amyl acetate | 123-92-2 | 61 | ester |
| 1173 | SAFC | 2-methyl-1-butanol | 137-32-6 | 56 | alcohol |
| 1184 | Fluka | methyl caproate | 106-70-7 | 74 | ester |
| 1186 | Aldrich | (R)-(-)-α-phellandrene | 99-83-2 | 91 | terpenes |
| 1187 | Fluka | 2-heptanone | 110-43-0 | 58 | ketone |
| 1211 | SAFC | 2-pentylfuran | 3777-69-3 | 81 | ethers |
| 1213 | Fluka | limonene | 138-86-3 | 68 | terpenes |
| 1218 | SAFC | butanoic acid, butyl ester | 109-21-7 | 71 | ester |
| 1220 | Aldrich | ethyl hexanoate | 123-66-0 | 88 | ester |
| 1234 | Fluka | 1,8-cineole | 470-82-6 | 81 | terpenes |
| 1234 | Aldrich | butyl 2-methylbutanoate | 15706-73-7 | 103 | ester |
| 1238 | Fluka | 1-pentanol | 71-41-0 | 42 | alcohol |
| 1250 | SAFC | acetoin | 513-86-0 | 43 | ketone |
| 1269 | SAFC | hexyl acetate | 142-92-7 | 61 | ester |
| 1276 | Aldrich | octanal | 124-13-0 | 57 | aldehyde |
| 1284 | Fluka | 2-heptanol | 543-49-7 | 45 | alcohol |
| 1287 | SAFC | isoamyl butyrate | 106-27-4 | 71 | ester |
| 1291 | Fluka | 2-octanone | 111-13-7 | 58 | ketone |
| 1295 | Fluka | p-cymene | 99-87-6 | 119 | terpenes |
| 1312 | SAFC | terpinolene | 586-62-9 | 121 | terpenes |
| 1316 | SAFC | hexyl propionate | 2445-76-3 | 57 | ester |
| 1319 | Supelco | 1-hexanol | 111-27-3 | 56 | alcohol |
| 1325 | SAFC | *trans*-2-hexenyl acetate | 2497-18-9 | 43 | ester |
| 1340 | Aldrich | 6-methyl-5-hepten-2-one | 110-93-0 | 43 | ketone |
| 1341 | SAFC | 3-methyl-1-pentanol | 589-35-5 | 56 | alcohol |
| 1352 | Fluka | *cis*/*trans* rose oxide | 16409-43-1 | 139 | terpenes |
| 1367 | SAFC | *trans*-3-hexenol | 928-97-2 | 56 | alcohol |
| 1369 | Aldrich | *cis*-3-hexen-1-ol | 928-96-1 | 67 | alcohol |
| 1369 | SAFC | heptyl acetate | 112-06-1 | 61 | ester |
| 1380 | SAFC | *trans*-2-hexen-1-ol | 928-95-0 | 54 | alcohol |
| 1382 | Fluka | 2-nonanone | 821-55-6 | 58 | ketone |
| 1385 | Aldrich | 2-octanol(IS) | 123-96-6 | 45 | alcohol |
| 1400 | Aldrich | 2-isopropyl-3-methoxypyrazine | 93905-03-4 | 137 | pyrazine |
| 1402 | SAFC | *trans*,*trans*-2,4-hexadienal | 142-83-6 | 65 | aldehyde |
| 1407 | SAFC | hexyl butyrate | 2639-63-6 | 71 | ester |
| 1410/1471 | Aldrich | *cis* and *trans* linalool oxide (Ox A+Ox B) | 60047-17-8 | 59 | terpenes |
| 1413 | SAFC | hexanoate butyl- | 626-82-4 | 99 | ester |
| 1418 | SAFC | hexyl 2-methylbutanoate | 10032-15-2 | 103 | ester |
| 1424 | Fluka | 1-heptanol | 111-70-6 | 57 | alcohol |
| 1424 | Fluka | acetic acid | 64-19-7 | 60 | acid |
| 1428 | Aldrich | ethyl caprylate | 106-32-1 | 88 | ester |
| 1430 | SAFC | *trans*-2-octenal | 2548-87-0 | 59 | aldehyde |
| 1462 | Fluka | sabinene hydrate | 80-56-8 | 93 | terpenes |
| 1465 | Fluka | 2-ethyl-1-hexanol | 104-76-7 | 57 | alcohol |
| 1471 | SAFC | 6-methyl-5-hepten-2-ol | 1569-60-4 | 95 | alcohol |
| 1472 | SAFC | octyl acetate | 103-09-3 | 61 | ester |
| 1475 | Aldrich | 2-sec-butyl-3-methoxypyrazine | 24168-70-5 | 138 | pyrazine |
| 1480 | SAFC | *trans*,*trans*-2,4-heptadienal | 4313-03-5 | 81 | aldehyde |
| 1491 | SAFC | citronellal | 106-23-0 | 71 | terpenes |
| 1497 | Aldrich | decanal | 112-31-2 | 111 | aldehyde |
| 1507 | Aldrich | theaspirane A | 36431-72-8 | 138 | terpenes |
| 1507 | Aldrich | theaspirane B | 36431-72-8 | 138 | terpenes |
| 1518 | SAFC | 2-isobutyl-3-methoxypyrazine | 24683-00-9 | 124 | pyrazine |
| 1520 | Aldrich | benzaldehyde | 100-52-7 | 106 | aldehyde |
| 1532 | Fluka | (-)-linalool | 78-70-6 | 71 | terpenes |
| 1532 | Aldrich | camphor | 76-22-2 | 81 | terpenes |
| 1560 | SAFC | nonyl acetate | 143-13-5 | 61 | ester |
| 1572 | Aldrich | 5-methylfurfural | 620-02-0 | 110 | aldehyde |
| 1590 | SAFC | *trans*-2-octen-1-ol | 18409-17-1 | 70 | alcohol |
| 1593 | Aldrich | terpinen-4-ol | 562-74-3 | 71 | terpenes |
| 1598 | Aldrich | hexanoic acid, hexyl ester | 6378-65-0 | 99 | ester |
| 1600 | Aldrich | undecylicaldehyde | 112-44-7 | 82 | aldehyde |
| 1615 | SAFC | *cis*-5-octen-1-ol | 64275-73-6 | 57 | alcohol |
| 1621 | Aldrich | decanoate <ethyl-> | 110-38-3 | 88 | ester |
| 1637 | Aldrich | 2-methyl-2,4-pentanediol | 107-41-5 | 59 | alcohol |
| 1638 | Aldrich | phenylacetaldehyde | 122-78-1 | 120 | aldehyde |
| 1642 | Aldrich | *trans*-caryophyllene | 87-44-5 | 91 | terpenes |
| 1656 | Fluka | acetophenone | 98-86-2 | 105 | ketone |
| 1673 | SAFC | 1-phenylethyl acetate | 93-92-5 | 104 | ester |
| 1685 | Fluka | 2-methylbutyric acid | 116-53-0 | 74 | acid |
| 1693 | Aldrich | α-humulene | 6753-98-6 | 93 | terpenes |
| 1695 | SAFC | dodecanal | 112-54-9 | 71 | aldehyde |
| 1698 | Fluka | (+)-α-terpineol | 98-55-5 | 59 | terpenes |
| 1723 | Aldrich | 1-decanol | 112-30-1 | 70 | alcohol |
| 1726 | Aldrich | α-citral | 141-27-5 | 84 | terpenes |
| 1730 | Fluka | methyl salicylate | 119-36-8 | 120 | ester |
| 1735 | Aldrich | geranyl acetate | 105-87-3 | 68 | terpenes |
| 1788 | SAFC | acetic acid, 2 phenylethyl ester | 103-45-7 | 104 | ester |
| 1791 | Fluka | myrtenol | 515-00-4 | 108 | alcohol |
| 1795 | SAFC | hexyl octanoate | 1117-55-1 | 145 | ester |
| 1811 | Bedoukian research, inc. | dihydro α-ionone | 31499-72-6 | 95 | terpenes |
| 1814 | SAFC | β-damascenone | 23726-93-4 | 121 | terpenes |
| 1815 | SAFC | geraniol | 106-24-1 | 41 | terpenes |
| 1833 | Fluka | α-ionone | 127-41-3 | 121 | terpenes |
| 1853 | SAFC | linalyl isovalerate | 1118-27-0 | 93 | terpenes |
| 1862 | Aldrich | benzyl alcohol | 100-51-6 | 108 | alcohol |
| 1862 | Aldrich | guaiacol | 90-05-1 | 109 | alcohol |
| 1880 | Fluka | hexanoic acid | 142-62-1 | 60 | acid |
| 1888 | Fluka | α-ionol | 25312-34-9 | 95 | terpenes |
| 1890 | Aldrich | 2-phenylethanol | 60-12-8 | 91 | alcohol |
| 1965 | Fluka | (-)-caryophyllene oxide | 1139-30-6 | 79 | terpenes |
| 1975 | Aldrich | β-ionone | 79-77-6 | 177 | terpenes |
| 2027 | Fluka | ethyl myristate | 124-06-1 | 88 | ester |
| 2100 | Fluka | octanoic acid | 124-07-2 | 60 | acid |
| 2102 | Aldrich | ethyl cinnamate | 4610-69-9 | 131 | ester |
| 2123 | Aldrich | methyl N-methylanthranilate | 85-91-6 | 104 | ester |
| 2162 | SAFC | geranic acid | 459-80-3 | 69 | terpenes |
| 2168 | SAFC | eugenol | 97-53-0 | 164 | alcohol |
| 2170 | Fluka | 1-tetradecanol | 112-72-1 | 97 | alcohol |
| 2180 | Aldrich | 2-methoxy-4-vinylphenol | 7786-61-0 | 164 | phenol |
| 2181 | Fluka | methyl anthranilate | 134-20-3 | 119 | ester |
| 2187 | SAFC | 2-aminoacetophenone | 551-93-9 | 120 | ketone |
| 2187 | SAFC | δ−decalactone | 705-86-2 | 99 | ketone |
| 2200 | Fluka | nonanoic acid | 112-05-0 | 60 | acid |
| 2243 | Fluka | decanoic acid | 334-48-5 | 60 | acid |
| 2345 | SAFC | methyl jasmonate | 42536-97-0 | 83 | ester |
| 2354 | Aldrich | *trans*-*trans*-farnesol | 16106-95-9 | 69 | terpenes |
| 2410 | Fluka | benzoic acid | 65-85-0 | 74 | acid |
| 2410 | SAFC | benzophenone | 119-61-9 | 105 | ketone |
| 2486 | SAFC | 5-(hydroxymethyl) furfural | 67-47-0 | 97 | aldehyde |
| 2566 | Carlo erba | vanillin | 99-83-2 | 151 | aldehyde |
| 2640 | SAFC | acetovanillon | 498-02-2 | 151 | ketone |
| 2691 | Fluka | myristic acid | 544-63-8 | 60 | acid |
| 2771 | SAFC | vanillylacetone | 122-48-5 | 137 | ketone |
| 2880 | Aldrich | palmitic acid | 57-10-3 | 60 | acid |
| 2061 | Fluka | epiglobulol | 88728-58-9 | 82 | terpenes |
| 2280 | synthesized | rotundone | 18374-76-0 | 147 | terpenes |

Table S 2: Experimental parameters of DHS sampling

| DHS | | TDU | |
| --- | --- | --- | --- |
| Incubation time (min) | 20 | Initial temperature (ºC) | 50 |
| Incubation temperature (ºC) | 40 | End temperature (ºC) | 300 |
| Agitator speed (rpm) | 500 | Rate (ºC/min) | 120 |
| Trapping volume (mL) | 1250 | Hold time (min) | 6 |
| Trapping flow (mL/min) | 100 | Transfer temperature (ºC) | 300 |
| Trapping temperature (ºC) | 40 | CIS | |
| Dry volume (mL) | 50 | Initial temperature (ºC) | 0 |
| Dry flow (mL/min) | 10 | End temperature (ºC) | 250 |
| Dry temperature (ºC) | 25 | Rate (ºC/s) | 12 |
|  |  | Hold time (min) | 10 |

Table S 3: TDU and CIS parameters for mSBSE analysis

| TDU | | CIS | |
| --- | --- | --- | --- |
| Initial temperature (ºC) | 30 | Initial temperature (ºC) | 0 |
| End temperature (ºC) | 220 | End temperature (ºC) | 250 |
| Rate (ºC/min) | 120 | Rate (ºC/s) | 12 |
| Hold time (min) | 10 | Hold time (min) | 10 |
| Transfer temperature (ºC) | 300 |  |  |

Table S 4: Experimental parameters of SPME sampling

| Sample preparation | | Fiber conditioning | |
| --- | --- | --- | --- |
| Incubation Temperature (ºC) | 30 | Bakeout Temperature (ºC) | 270 |
| Incubation Time (min) | 5 | Pre bakeout time (min) | 4.50 |
| Agitator speed (rpm) | 500 | Bakeout Penetration (mm) | 67 |
| Desorption time (min) | 4 |  |  |
| Desorption Temperature (ºC) | 250 |  |  |

Table S 5: Optimization of GCxGC separation condition. Results of experiment 6, 12, 25 or 26 were not used because of the heavily wrap round

| Exp | Column flow (mL/min) | Temp program (ºC/min) | 2nd oven temp offset (ºC) | Modulation temp offset (ºC) | Modulation time (s) | Hot pulse time (s) | Median NND (s) |
| --- | --- | --- | --- | --- | --- | --- | --- |
| **1** | 1.6 | 7 | 1 | 10 | 4 | 0.6 | 4.03 |
| **2** | 1.6 | 3 | 1 | 10 | 8 | 1.2 | 8.22 |
| **3** | 1.2 | 5 | 3 | 10 | 4 | 1 | 8.01 |
| **4** | 0.8 | 7 | 5 | 6 | 4 | 0.6 | 4.02 |
| **5** | 1.6 | 7 | 1 | 10 | 8 | 2.8 | 8.00 |
| **6** | 0.8 | 3 | 5 | 6 | 4 | 1.4 |  |
| **7** | 0.8 | 5 | 1 | 6 | 8 | 2.8 | 8.00 |
| **8** | 1.2 | 7 | 5 | 6 | 8 | 2.8 | 8.00 |
| **9** | 1.6 | 3 | 5 | 6 | 8 | 2 | 8.28 |
| **10** | 0.8 | 3 | 1 | 10 | 6 | 2.1 | 12.01 |
| **11** | 1.2 | 5 | 5 | 8 | 6 | 0.9 | 6.02 |
| **12** | 1.6 | 3 | 1 | 8 | 4 | 1.4 |  |
| **13** | 0.8 | 7 | 5 | 10 | 4 | 1.4 | 4.02 |
| **14** | 1.6 | 7 | 5 | 8 | 4 | 1 | 4.07 |
| **15** | 1.2 | 3 | 3 | 8 | 8 | 2.8 | 12.18 |
| **16** | 1.6 | 5 | 3 | 6 | 6 | 2.1 | 6.03 |
| **17** | 1.6 | 7 | 5 | 10 | 8 | 1.2 | 8.00 |
| **18** | 1.6 | 3 | 5 | 10 | 6 | 2.1 | 12.04 |
| **19** | 0.8 | 7 | 1 | 10 | 8 | 1.2 | 8.00 |
| **20** | 0.8 | 5 | 1 | 8 | 4 | 0.6 | 8.00 |
| **21** | 1.2 | 3 | 1 | 6 | 6 | 1.5 | 12.04 |
| **22** | 1.6 | 7 | 1 | 6 | 8 | 1.2 | 8.00 |
| **23** | 0.8 | 7 | 3 | 8 | 6 | 1.5 | 6.00 |
| **24** | 1.2 | 7 | 1 | 6 | 4 | 1.4 | 4.01 |
| **25** | 0.8 | 3 | 5 | 10 | 4 | 0.6 |  |
| **26** | 1.6 | 3 | 3 | 6 | 4 | 0.6 |  |
| **27** | 0.8 | 5 | 5 | 10 | 8 | 2 | 8.00 |
| **28** | 0.8 | 3 | 3 | 6 | 8 | 1.2 | 8.12 |

Table S 6: Model fitting summary

| RSquare | 1 |
| --- | --- |
| RSquare Adj | 0.999997 |
| Root Mean Square Error | 0.0042 |
| Mean of Response | 7.629552 |
| Observations (or Sum Wgts) | 24 |

Table S 7: Model effect summary

| **Source** | **LogWorth** |  | **PValue** |
| --- | --- | --- | --- |
| temp program(3,7) | 6.238 |  | 0.00000 |
| modulation time*temp program | 5.940 |  | 0.00000 |
| temp program*temp program | 5.466 |  | 0.00000 |
| modulation time*modulation time | 5.315 |  | 0.00000 |
| modulation temp*modulation temp | 5.046 |  | 0.00001 |
| temp program*hot pulse time | 5.031 |  | 0.00001 |
| column flow(0.8,1.6) | 4.909 |  | 0.00001 |
| column flow*2nd oven temp offset | 4.873 |  | 0.00001 |
| hot pulse time(0.15,0.35) | 4.845 |  | 0.00001 |
| modulation time(4,8) | 4.771 |  | 0.00002 |
| modulation temp*hot pulse time | 4.488 |  | 0.00003 |
| 2nd oven temp offset*modulation temp | 4.363 |  | 0.00004 |
| hot pulse time*hot pulse time | 3.959 |  | 0.00011 |
| modulation temp(6,10) | 3.451 |  | 0.00035 |
| 2nd oven temp offset*hot pulse time | 3.365 |  | 0.00043 |
| modulation time*hot pulse time | 3.243 |  | 0.00057 |
| column flow*modulation temp | 3.106 |  | 0.00078 |
| column flow*modulation time | 3.086 |  | 0.00082 |
| column flow*hot pulse time | 2.902 |  | 0.00125 |
| 2nd oven temp offset(1,5) | 2.801 |  | 0.00158 |
| modulation time*2nd oven temp offset | 2.532 |  | 0.00293 |

Table S 8: Parameter Estimates

| **Term** | **Estimate** | **Std Error** | **t Ratio** | **Prob>\|t\|** |
| --- | --- | --- | --- | --- |
| Intercept | 6.8892815 | 0.005133 | 1342.1 | <.0001* |
| column flow(0.8,1.6) | -0.357395 | 0.001255 | -284.8 | <.0001* |
| modulation time(4,8) | -0.568177 | 0.002339 | -243.0 | <.0001* |
| 2nd oven temp offset(1,5) | -0.032752 | 0.001304 | -25.13 | 0.0016* |
| temp program(3,7) | -3.566881 | 0.002712 | -1315 | <.0001* |
| modulation temp(6,10) | 0.0809492 | 0.001523 | 53.16 | 0.0004* |
| hot pulse time(0.15,0.35) | 0.6585843 | 0.002489 | 264.57 | <.0001* |
| column flow*modulation time | -0.053087 | 0.001521 | -34.89 | 0.0008* |
| modulation time*modulation time | 1.5732576 | 0.003463 | 454.31 | <.0001* |
| column flow*2nd oven temp offset | -0.525036 | 0.001922 | -273.1 | <.0001* |
| modulation time*2nd oven temp offset | -0.027709 | 0.001504 | -18.42 | 0.0029* |
| modulation time*temp program | 2.8814911 | 0.003087 | 933.53 | <.0001* |
| temp program*temp program | 2.3224086 | 0.004293 | 540.92 | <.0001* |
| column flow*modulation temp | 0.0902685 | 0.002527 | 35.72 | 0.0008* |
| 2nd oven temp offset*modulation temp | 0.3464299 | 0.002281 | 151.86 | <.0001* |
| modulation temp*modulation temp | -1.105736 | 0.003314 | -333.6 | <.0001* |
| column flow*hot pulse time | 0.0402426 | 0.001425 | 28.24 | 0.0013* |
| modulation time*hot pulse time | 0.1404141 | 0.003357 | 41.82 | 0.0006* |
| 2nd oven temp offset*hot pulse time | -0.075921 | 0.001577 | -48.15 | 0.0004* |
| temp program*hot pulse time | -0.739247 | 0.002257 | -327.6 | <.0001* |
| modulation temp*hot pulse time | -0.339576 | 0.001936 | -175.4 | <.0001* |
| hot pulse time*hot pulse time | -0.260668 | 0.002732 | -95.40 | 0.0001* |

**Fig S 1** Prediction of median NND by the Box-Behnken surface response model under optimized oven condition


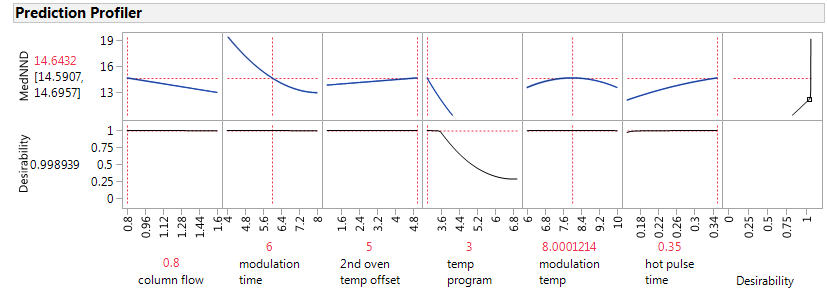


**Fig S 2** MS similarity (matching score) distribution


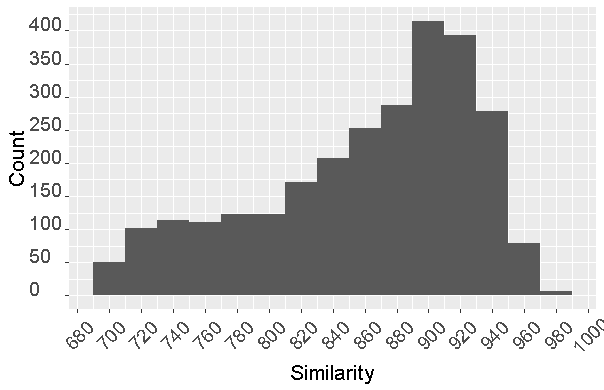

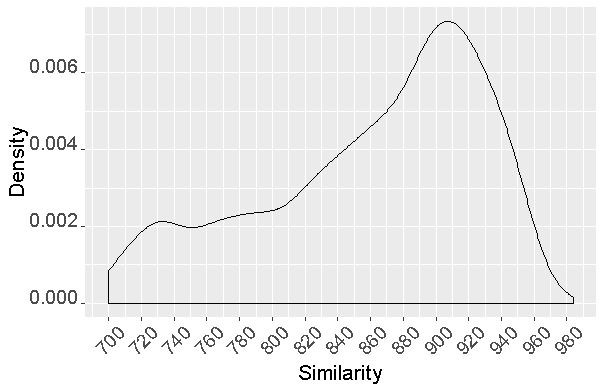


**Fig S 3** Over deconvolution of peak 1-phenylethyl acetate. a) 2d chromatogram of peak 1-phenylethyl acetate, b) constructed mass spectrum of peak 1-phenylethyl acetate, c) library mass spectrum of 1-phenylethyl acetate (Nist), d) constructed mass spectrum of unknown peak


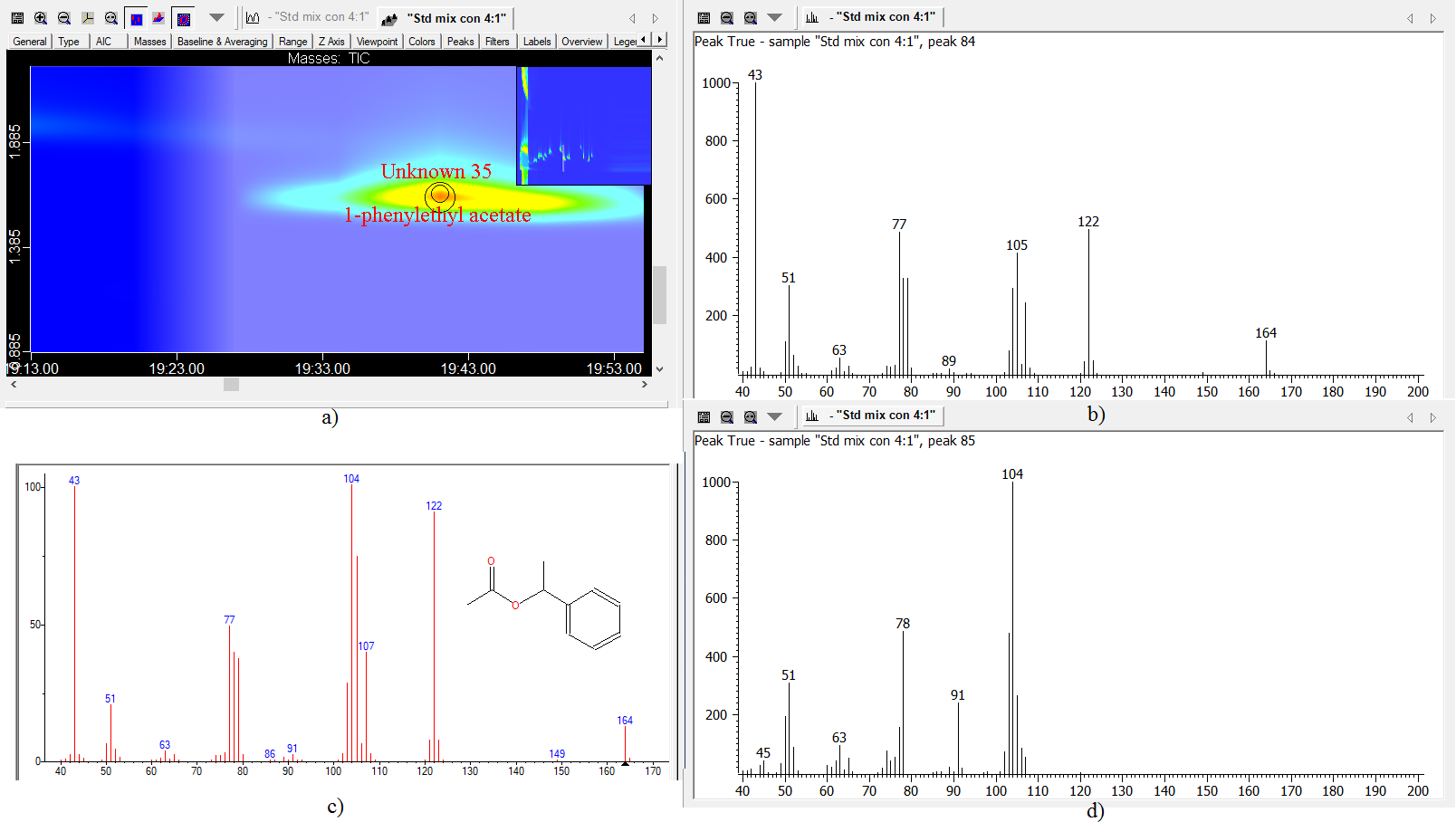


**Fig S 4** Comparison of the aligned peak number by chemical classes for applying different sampling techniques on pooled red wine


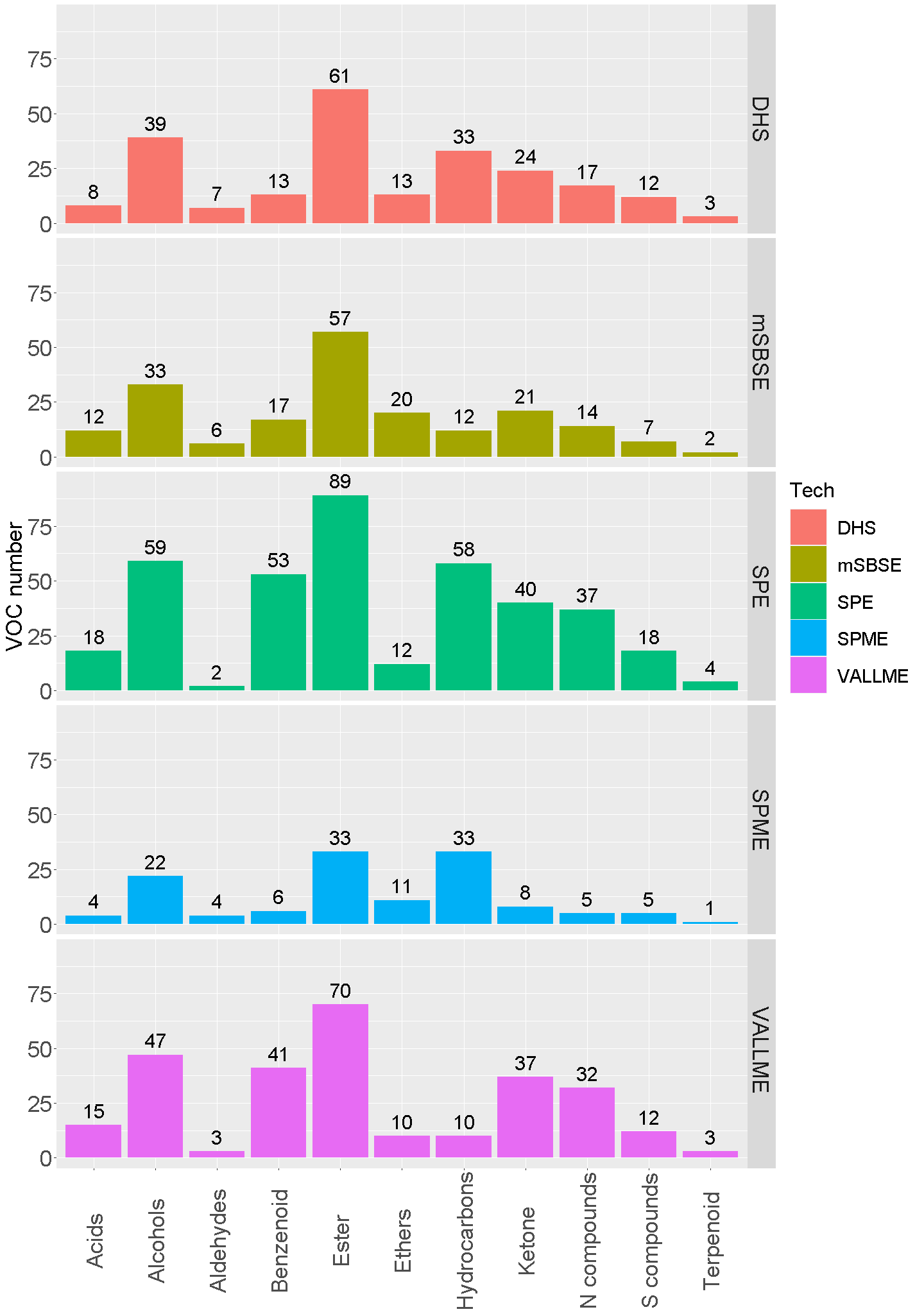


**Fig S 5** Comparison of the aligned peak number by chemical classes for applying different sampling techniques on pooled cider


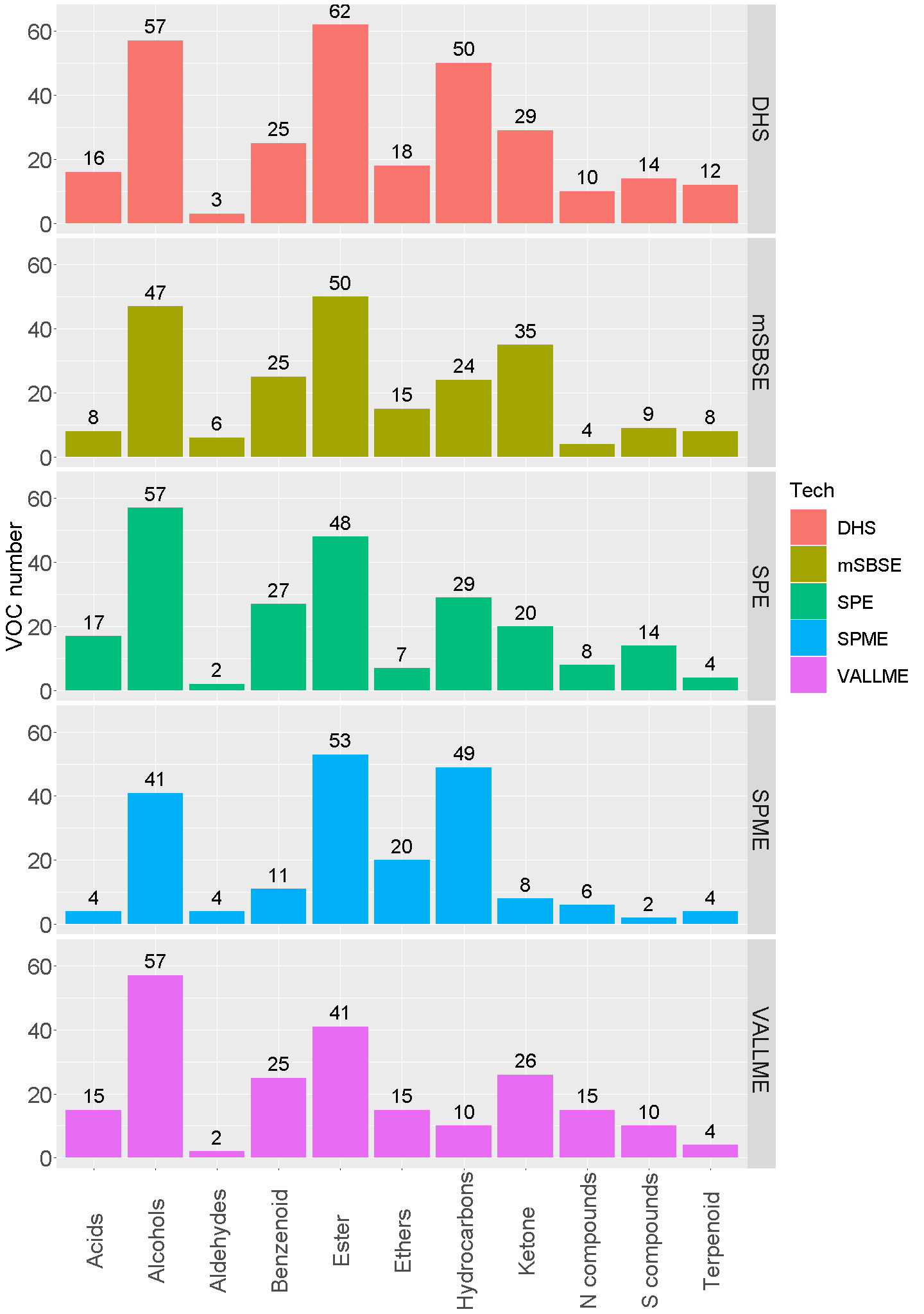


**Fig S 6** Comparison of the aligned peak number by chemical classes for applying different sampling techniques on pooled white wine


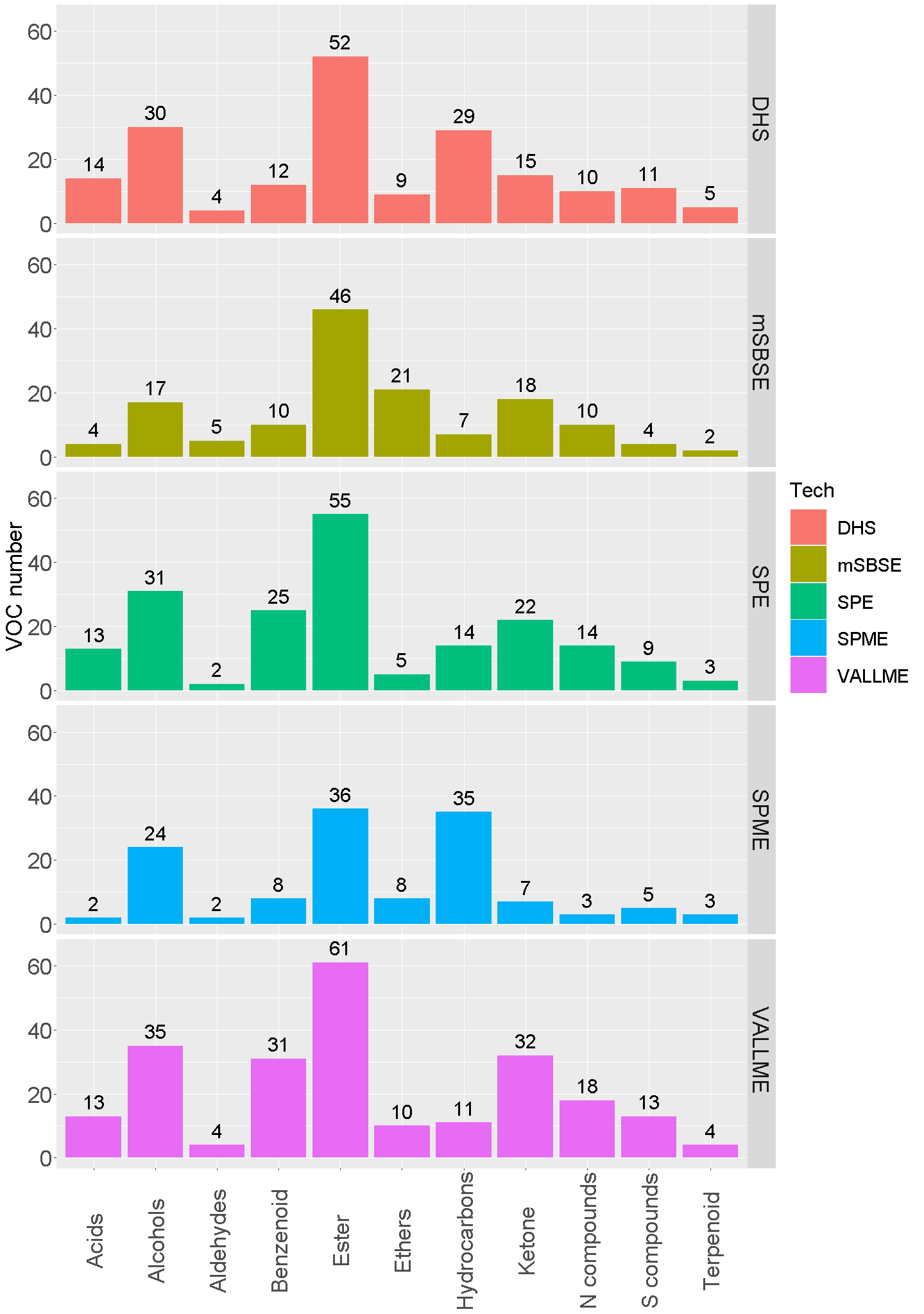


**Fig S 7** mSBSE insufficient TD


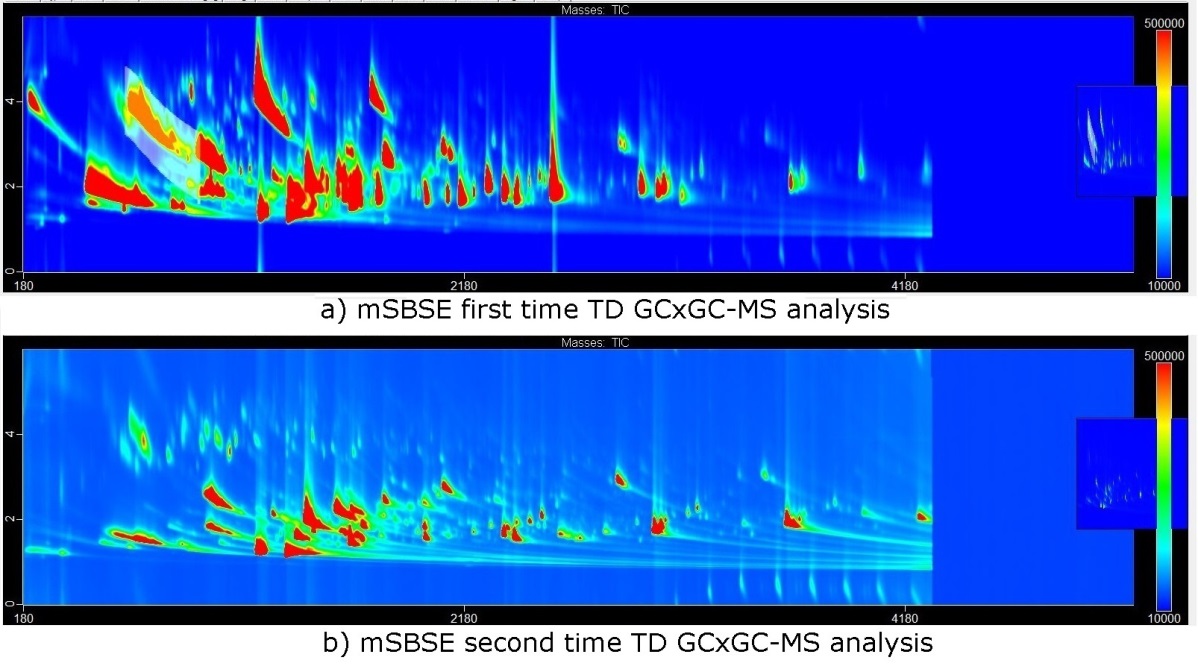


**Fig S 8** Heatplot for fermentative aromatic compounds determination with different sampling techniques on pooled beer


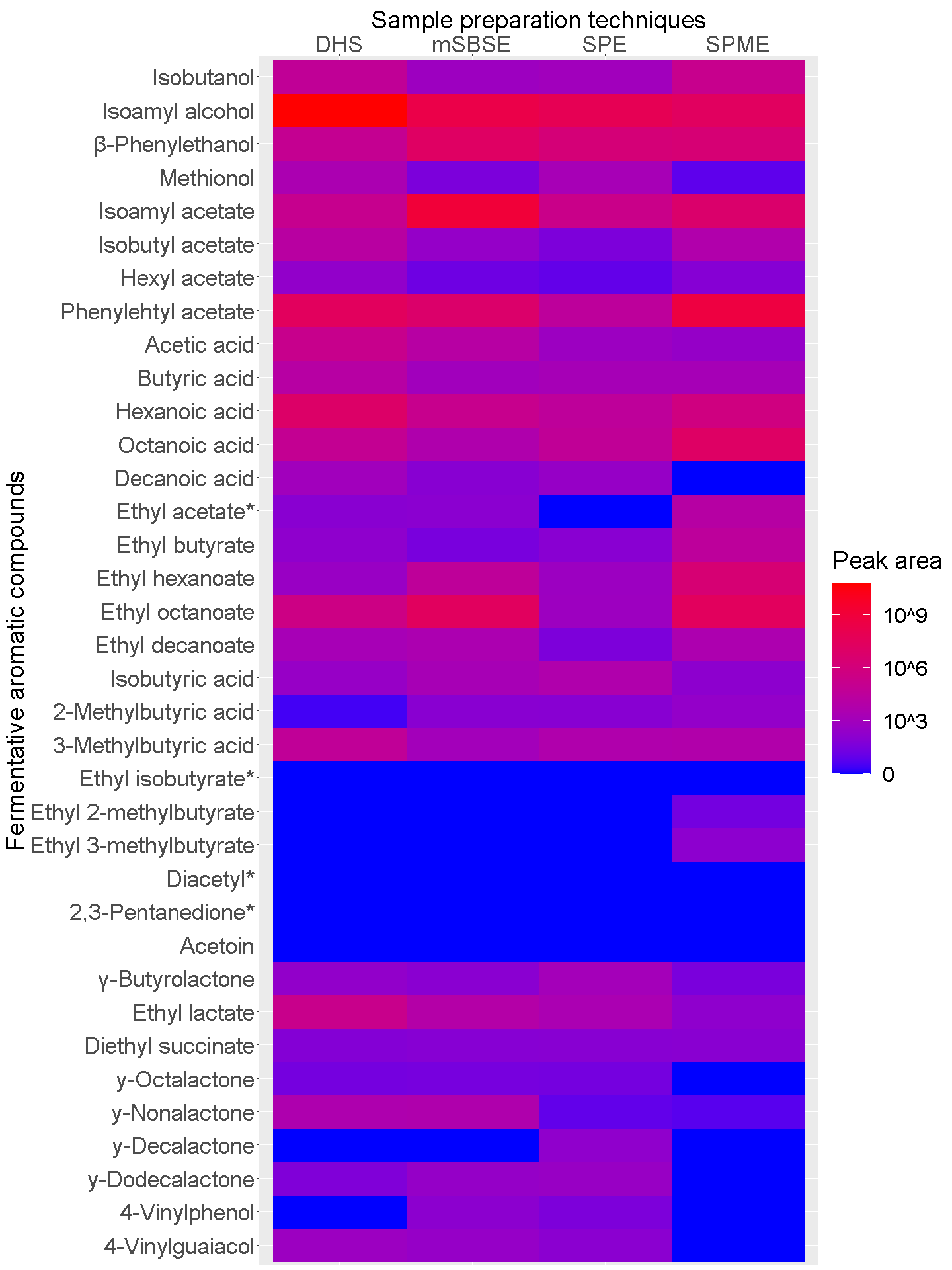


**Fig S 9** Heatplot for fermentative aromatic compounds determination with different sampling techniques on pooled red wine


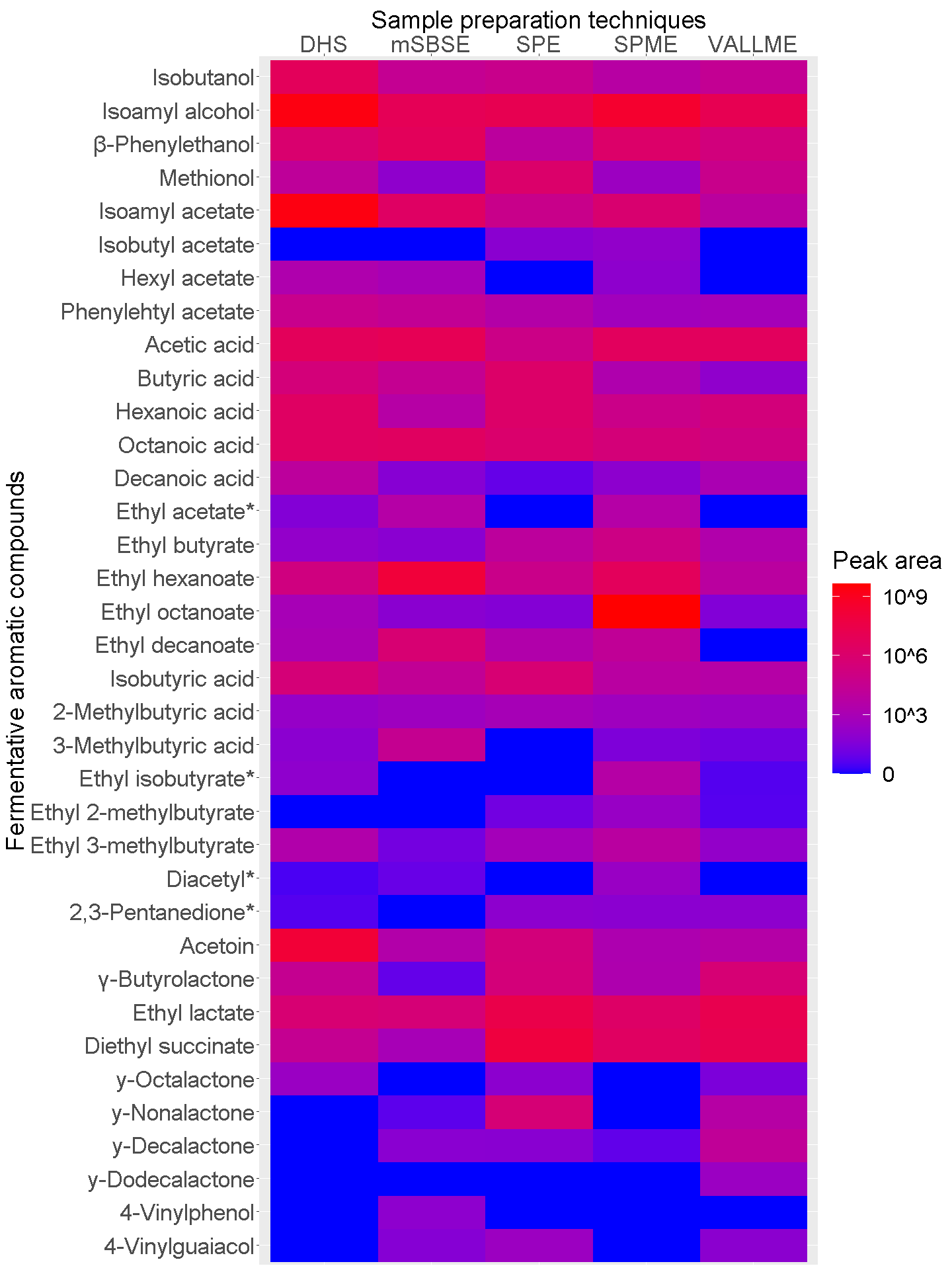


**Fig S 10** Heatplot for fermentative aromatic compounds determination with different sampling techniques on pooled cider


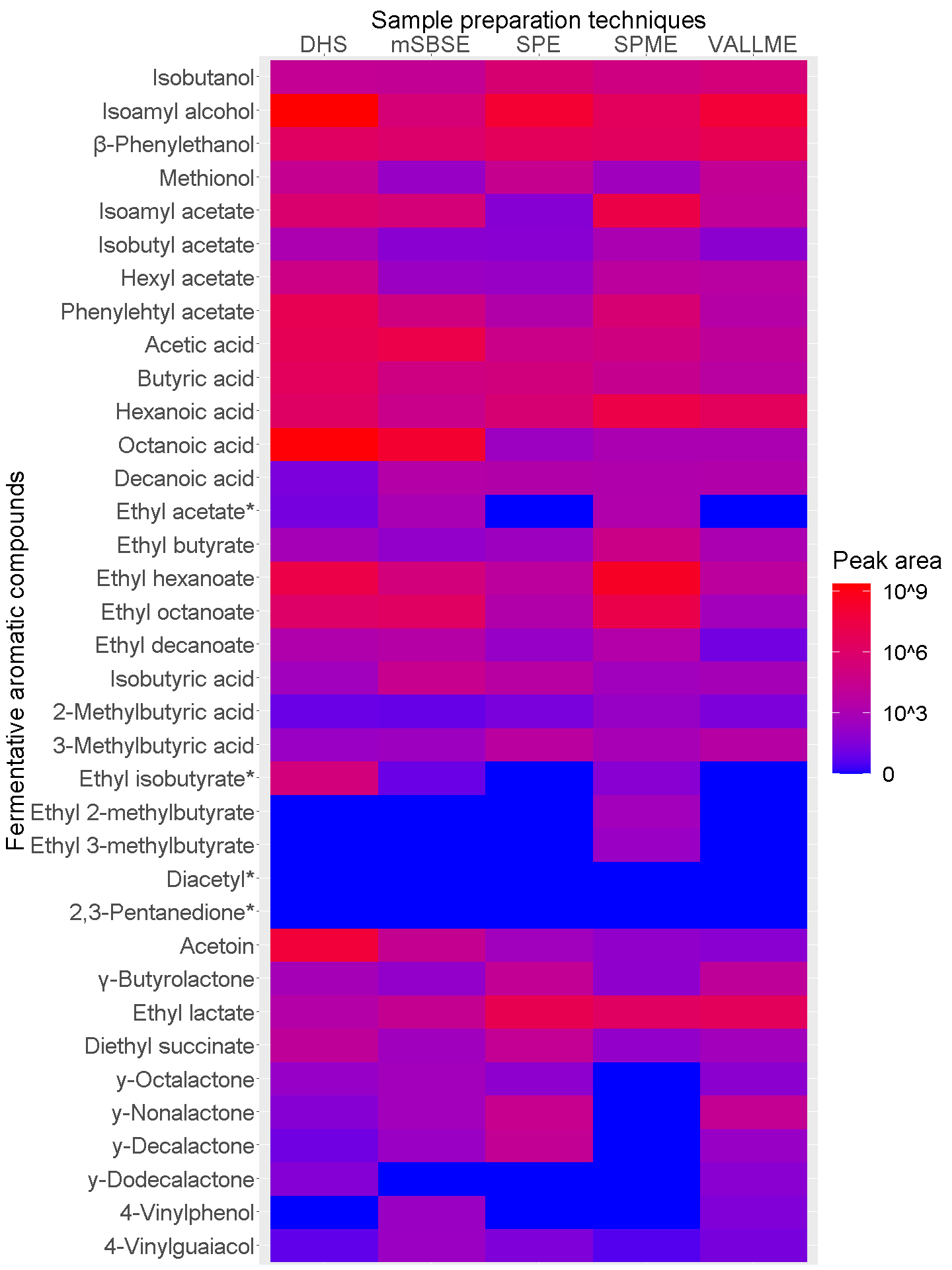

Supplement: Supplementary file 1 — Supplementary file1 (DOCX 671 kb) [file 11306_2020_1718_MOESM1_ESM.docx]
